# Draft Genome Sequence of an Escherichia coli Strain Harboring *bla*_CTX-M-115_, *bla*_CMY-2_, Aminoglycoside, Tetracycline, and Sulfonamide Resistance Genes, Isolated from a Costa Rican Wastewater Treatment Plant

**DOI:** 10.1128/MRA.01015-19

**Published:** 2020-01-02

**Authors:** Kenia Barrantes, Luz María Chacón, Eric Morales, Lisbeth Ramírez-Carvajal

**Affiliations:** a Sección Infección-Nutrición, Instituto de Investigaciones en Salud (INISA), Universidad de Costa Rica, San José, Costa Rica; b Laboratorio Nacional de Servicios Veterinarios (LANASEVE), Servicio Nacional de Salud Animal (SENASA), Ministerio de Agricultura y Ganadería, Heredia, Costa Rica; University of Maryland School of Medicine

## Abstract

We report the draft genome sequence of the multidrug-resistant Escherichia coli strain PTA A1517-5, isolated from a wastewater treatment plant in Costa Rica. The genome consists of 4,927,375 bp with a GC content of 50.57% and a total of 4,853 genes. This strain harbors *bla*_CTX-M-115_, *bla*_CMY-2_, aminoglycoside, tetracycline, and sulfonamide resistance genes.

## ANNOUNCEMENT

Escherichia coli is a well-known and frequently used indicator of fecal pollution. This bacterium has also been shown to be a reservoir of antimicrobial resistance (AMR) genes. Detection of E. coli harboring AMR genes could provide information on the occurrence and spread of antibiotic resistance in the environment (1–5).

E. coli strain PTA A1517-5 was isolated from a wastewater sample which was collected from the effluent of a domestic wastewater treatment plant (WWTP) located in Alajuela, Costa Rica. E. coli organisms were enumerated from the WWTP effluent using the most probable number (MPN) technique according to American Public Health Association (APHA) guidelines (6). Briefly, the wastewater sample was inoculated into lauryl tryptose broth (Oxoid) and incubated at 35.0°C for 48 h. All tubes testing positive after the incubation period were inoculated into EC-MUG broth (Oxoid). After a 24-h incubation period at 44.5°C, tubes with a positive reaction were inoculated onto MacConkey agar plates (Oxoid) and incubated at 35°C for 24 h. The E. coli strain was identified using biochemical (API20E; BioMérieux) and molecular (16S rRNA) methods (7).

The antibiotic susceptibility profile was assessed according to 2014 CLSI guidelines (15). The E. coli strain showed resistance to amoxicillin (AML), cephalothin (KF), cefazolin (KZ), cefotaxime (CTX), tetracycline (TE), gentamicin (CN), and trimethoprim-sulfamethoxazole (SXT).

After biochemical identification, a single colony of the E. coli strain was picked and further grown in Trypticase soy broth (Oxoid) at 35°C for 18 to 24 h. Genomic DNA was extracted from the E. coli strain using a DNeasy blood and tissue kit (Qiagen). DNA quality and quantity were measured using a NanoDrop instrument (Thermo Fisher, Waltham, MA, USA) and a Quantus fluorometer (Promega, Wisconsin, USA). A dilution of 0.2 ng/μl of genomic DNA was used to prepare libraries with a Nextera XT DNA library prep kit (Illumina, San Diego, CA, USA) following the manufacturer’s instructions. The library was sequenced on an Illumina MiSeq instrument using a paired-end (2 × 250-bp) protocol.

The paired-end reads’ trimming quality was assessed using FastQC v0.11.5, and it was conducted with the seqtk toolkit (8) using the parameters q = 0.1 and L = 200. A total of 1,895,908 reads were obtained after trimming. Reads were assembled *de novo* using SPAdes v3.13.0 (9) with default settings and included a built-in BayesHammer read error correction tool. All contigs smaller than 500 bp were removed.

The genome was annotated using the NCBI Prokaryotic Genome Annotation Pipeline (PGAP) v4.8 (10).

The E. coli PTA A1517-5 draft genome sequence consists of 4,927,375 bp in 82 contigs with a GC content of 50.57%, an *N*_50_ value of 210,703 bp, a total of 4,853 genes, and a genome coverage of 31.2×.

AMR genes were queried in the ResFinder (11), PATRIC (12), and CARD databases (13) using default parameters.

The antibiotic resistance phenotype of strain PTA A1517-5 was consistent with the presence of *bla*_CTX-M-115_, *bla*_CMY-2_, aminoglycoside, tetracycline, and sulfonamide resistance genes.

**FIG 1:**
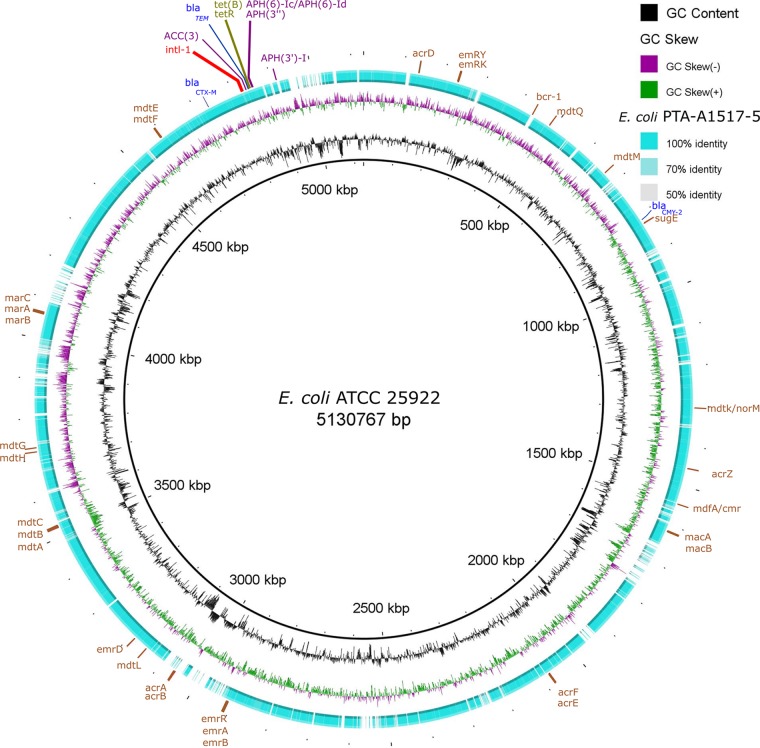
Genome alignment of E. coli strain PTA A1517-5 using the BLAST Ring Image Generator (BRIG) software (14). Multidrug efflux system genes are denoted in brown letters (*emrY*, *emrK*, *Bcr-1*, *mdtQ*, *mdtM*, *sugE*, *mdtK, norM*, *acrZ*, *mdfA/cmr*, *macA*, *macB*, *acrF*, *acrE*, *emrR*, *emrA*, *emrB*, *acrA*, *acrB*, *mdtL*, *emrD*, *mdtA*, *mdtB*, *mdtC*, *mdtH*, *mdtG*, *marA*, *marC*, *marB*, *mdtE*, and *mdtF*). Beta-lactamase genes (*bla*_TEM_, *bla*_CTX-M-115_, and *bla*_CMY-2_) are denoted in blue letters. Class 1 integron genes (*intI-1*, *dfrA12*, *gcuF*, *aadA2*, *qacEdelta1*, and *sul1*) are marked with red letters. Aminoglycoside resistance genes [*ACC(3)*, *APH(3ʺ)*-*I*, *APH(3ʺ)*, *APH(6)-Ic*, and *APH(6)-Id*] are denoted in purple letters, and tetracycline resistance genes [*tet(B)* and *tetR*] are marked with green letters. The genome of E. coli strain ATCC 25922 was used as the reference (GenBank accession no. CP009072).

BLAST Ring Image Generator (BRIG) (14) was used to visualize the coding sequence identity between the E. coli strain PTA A1517-5 and the reference E. coli strain ATCC 25922 (Fig. 1). Genes related to AMR are indicated in Fig. 1.

### Data availability.

This whole-genome shotgun sequencing project has been deposited in DDBJ/ENA/GenBank under the accession no. VMHG00000000. The version described in this paper is version VMHG02000000. The reads were deposited in the Sequence Read Archive (SRA) under accession no. PRJNA556251.
